# Cross-Cultural Differences in Public Discourse on COVID-19 Vaccination in the United States and South Korea: Cross-Sectional Analysis Using Natural Language Processing

**DOI:** 10.2196/84791

**Published:** 2026-03-05

**Authors:** Sangpil Youm, Sou Hyun Jang, Haewoon Kwak, Jaeyoung Choi, Yong Jeong Yi

**Affiliations:** 1 Department of Computer and Information Science and Engineering University of Florida Gainesville, FL United States; 2 Department of Sociology Korea University Seoul Republic of Korea; 3 Luddy School of Informatics, Computing, and Engineering Indiana University Bloomington, IN United States; 4 Aml&Regtech Team Naver Financial Seongnam Republic of Korea; 5 Department of Applied Artificial Intelligence College of Computing & Informatics Sungkyunkwan University Seoul Republic of Korea

**Keywords:** COVID-19 vaccine, natural language processing, topic detection, sentiment analysis, temporal dynamics, health communication

## Abstract

**Background:**

The COVID-19 vaccine was introduced as a crucial tool to combat the pandemic. However, concerns about its effectiveness, side effects, and misinformation spread remain. Prior research largely relied on survey-based approaches with limited populations. To address these limitations, social media offers a broader, more naturalistic lens into public discourse on COVID-19 vaccination. Accordingly, our study leverages social media data to identify factors shaping vaccine-related information needs, perceptions, and communication dynamics.

**Objective:**

This study investigated public discourse about COVID-19 vaccines on community-driven question-and-answer sites in the United States (Quora; Quora, Inc) and South Korea (Naver Knowledge-iN; Naver Corp) to identify cross-national similarities and differences in vaccine-related information needs, sentiment patterns, and public perceptions over time.

**Methods:**

We analyzed publicly available COVID-19 vaccine–related questions and answers posted between June 27, 2020, and June 27, 2021, on 2 community-driven question-and-answer platforms: Quora (United States) and Naver Knowledge-iN (South Korea). After preprocessing and sample-size matching, the dataset included 3952 question-answer pairs per platform, with one community-selected (most upvoted) answer analyzed per question. Natural language processing (NLP) techniques were applied for topic classification and sentiment analysis. Questions were categorized using a hybrid topic modeling approach combining Latent Dirichlet Allocation (LDA) and Top2Vec, identifying 5 topics on Quora and 7 topics on Naver Knowledge-iN. Answer sentiments were classified using an ensemble of Bidirectional Encoder Representations from Transformers (BERT; Google LLC)– and Efficiently Learning an Encoder that Classifies Token Replacements Accurately (ELECTRA; Google LLC)–based transformer models, and temporal sentiment trends were examined using monthly aggregation.

**Results:**

Five shared information needs emerged, including effects of vaccines, variants, government policy, visiting overseas, and different vaccines, while South Korea uniquely exhibited vaccination appointments (711/3952, 18%) and school and education (513/3592, 13%). Negative sentiment predominated in US (Quora) answers across 4 of 5 topics, whereas positive sentiment exceeded 50% (498/790, 337/474, 367/592, 218/316, 348/553, 562/711, and 364/513) across all 7 topics on Naver Knowledge-iN. Temporally, US sentiment exhibited multiple positive-negative crossovers, whereas Korean sentiment stabilized toward positivity after February 2021, coinciding with the national vaccine rollout. Question-answer sentiment pairs showed contrasting interaction patterns, including negative-negative pairs dominated in the United States (eg, 504/978, 51.5% for different vaccines), while in South Korea, positive-positive and negative-positive pairs accounted for more than 63% (498/790, 337/474, 367/592, 218/316, 348/553, 562/711, and 364/513) of interactions in 7 topics, with positive-positive pairs most prevalent in 6 of 7 topics, except for variants.

**Conclusions:**

Public perceptions of COVID-19 vaccines and related information needs differ between the 2 countries, shaped by cultural context, trust in government, and information-seeking environments. Analysis of social question and answer data from the 2 countries reveals shared information needs but divergent sentiment patterns. These findings highlight the value of social media data for public health research and the need for culturally and platform-specific communication strategies.

## Introduction

Since the recognition of COVID-19 in December 2019, many efforts have been made to control it. Before the development and distribution of the COVID-19 vaccine, the World Health Organization (WHO) and the Centers for Disease Control and Prevention (CDC) recommended passive preventive measures like mask-wearing, hand hygiene, and social distancing [1,2]. The COVID-19 vaccine, released in 2020 [3], was anticipated to be a pivotal tool in ending the pandemic. However, the scientific evidence supporting its effectiveness remains limited, and concerns about potential side effects persist [4,5]. Furthermore, misinformation about the vaccine has fueled public uncertainty and apprehension [6-8], contributing to vaccine hesitancy [4,9-11].

These concerns were reflected in real-world vaccination uptake, which varied across populations and national contexts. Despite the availability of COVID-19 vaccines in both the United States and Korea, vaccination rates stalled at some point, with 68.7% fully vaccinated in the United States [12] and 85.6% in Korea [13] as of October 2022. Public perceptions and intentions toward vaccination varied significantly in both countries [14-17].

In the United States, numerous studies have focused on vaccine hesitancy, analyzing factors like sociodemographic characteristics [5,14,15,18,19]. Vaccination hesitancy was notably higher among those experiencing social injustices, including race and religious discrimination, with hesitancy rates at 68.9% for non-Hispanic Whites, 74% for African Americans, and 59.5% for Hispanics or Latinos [15]. Lower-income Americans showed less fear of COVID-19 and less trust in vaccines, while higher-income Whites were more willing to get vaccinated [18,20]. Political affiliations further highlighted disparities, with Republicans exhibiting the highest vaccine hesitancy [21,22]. Racial injustice exhibited higher vaccine hesitancy due to limited access to information and greater mistrust in the government [19,23].

Taken together, existing studies have primarily relied on self-reported online surveys to characterize vaccine hesitancy, emphasizing the roles of personal attributes, demographic characteristics, and political orientations, while offering limited insight into how concerns and information needs are expressed in natural settings [24,25]. However, many studies are limited by focusing on specific populations rather than the general public [26]. Survey data limitations can be mitigated by analyzing public opinions expressed on social media [27]. Social media is a natural setting for understanding public perceptions and information needs regarding the COVID-19 vaccine, potentially uncovering new factors influencing vaccine uptake [28].

To contextualize these vaccine-related discussions, social media serves as a central channel for exchanging health information, enabling users to share advice, knowledge, and experiences [27,29] while also playing a critical role in public health communication during health crises such as Hemagglutinin Type 1 and Neuraminidase Type 1 (H1N1), Middle East Respiratory Syndrome (MERS), and the COVID-19 pandemic [30,31].

During the COVID-19 pandemic, social media significantly impacted information sharing and vaccine hesitancy [32-35]. These studies underscore social media’s role in shaping public perceptions and providing social and informational support, fostering a sense of connectedness during lockdowns [36,37].

Although social media offers many benefits, previous studies have suggested its potential risks associated with the spread of misinformation, which may cause severe consequences [33,38]. In the case of vaccine information, negative content has been widely disseminated through social media channels, contributing to vaccine hesitancy [39-41].

On Parler (Parler LLC), conservative-leaning hashtags like #NoCovidVaccine have supported negative vaccine perceptions [42]. YouTube’s (Google LLC) user-generated content often includes misinformation due to limited regulations, and its recommendation systems promote conspiracy theories [43].

On Twitter (X Corp), sentiment toward vaccines varied by country; Switzerland had more positive tweets (11,621/16,268, 71.43%) than negative tweets, but the United States had only 10.48% positive tweets [44]. Only a small portion (< 10%, 9/107) of the most influential profiles engaging in the COVID-19 vaccine conversations originated from the medical professionals, indicating the need for public health stakeholders to address misinformation on social media [42,45].

Among these platforms, community-driven question and answer (Q&A) sites stand out as valuable online resources. Q&A sites like Quora (Quora, Inc) in the United States and Naver Knowledge-iN (Naver KiN; Naver Corporation) in South Korea (hereafter Korea) allow users to express their health information needs in natural language while fostering a sense of community [46]. During the COVID-19 pandemic, Q&A sites served as forums for discussing vaccines, offering valuable insights into public information needs. Understanding these needs and vaccine perceptions is crucial for creating effective vaccination campaigns [9,47]. To achieve this understanding, a strategic approach and the use of information and communication technology are essential [9].

Though not fully representative of Western and Eastern countries, the United States and Korea are frequently compared in health communication studies due to their distinct cultural traits, highlighting regional differences [48]. Throughout the COVID-19 pandemic, these countries exhibited disparities in vaccine-related aspects such as case proportions, quarantine regulations, and vaccine availability [49,50]. Nevertheless, there are few studies examining vaccine information needs and perceptions across multiple countries [51,52].

Consequently, we investigated the information needs, perceptions, and attitudes toward the COVID-19 vaccine expressed on social media by people in the United States and Korea. Our findings emphasize the complexity of public perceptions of vaccines and the critical need for targeted public health communication strategies. The unique information needs and varying sentiments across topics and Q&A pairs over time in both the United States and Korea illustrate the nuanced challenges faced by public health initiatives. Addressing those challenges through targeted, adaptive, and culturally aware communication efforts is essential for overcoming vaccine hesitancy, enhancing public trust, and ultimately achieving herd immunity against COVID-19.

## Methods

### Overview

This study conducted a cross-sectional analysis of vaccine-related questions and associated information needs on Quora and Naver KiN between June 2020 and June 2021. We followed the STROBE (Strengthening the Reporting of Observational Studies in Epidemiology; Multimedia Appendix 1) reporting guidelines. This study design is aligned with recent cross-sectional analyses of social media use for health-related issues [53].

To examine the perceptions and information needs of Q&A site users regarding the COVID-19 vaccine, we collected data from Q&A sites with representative US and Korean user bases. For this purpose, we chose Quora, a US platform hosting millions of questions and answers [54], and Naver KiN, the foremost social Q&A site in Korea [55]. Data were collected using Selenium (Software Freedom Conservancy, Inc), an open-source framework that allows automatic interaction with web browsers using Python 3.8.5 (Python Software Foundation) [56]. We set intervals during data collection to avoid overusing the service. For each question, we collected the URL, upload date, and answers. The data collection ran from June 27, 2020, to June 27, 2021, based on the upload dates for both websites, and the inclusion criteria were questions and answers pertinent to COVID-19 and any morphological form of the keyword “vaccine” (in Korean,*백신*), such as “vaccines” and words containing “vaccine.” By applying those criteria and excluding unanswered questions, we identified and subsequently removed questions falling outside our specified criteria.

**Figure 1:**
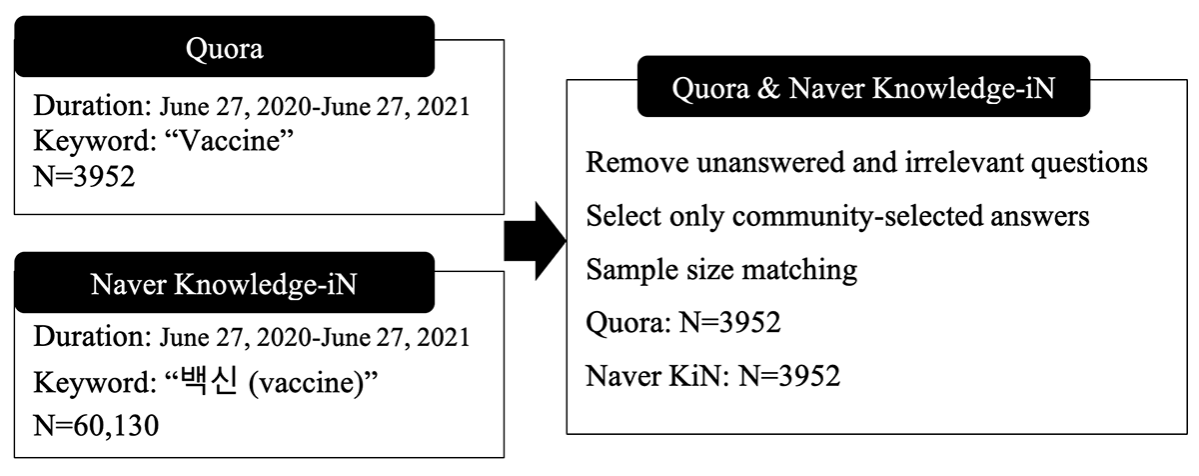
Data preprocessing. Data from Quora and Naver Knowledge-iN (Naver KiN) were collected between June 27, 2020, and June 27, 2021, using the keyword “Vaccine” (in Korean, 백신). After preprocessing, the Naver KiN data were randomly sampled to match the Quora sample size per platform (N= 3952).

As a result, after sampling the data, we collected 3952 sets of questions and answers from Quora and Naver KiN, as depicted in Figure 1. Rather than analyzing all the answers to the questions, we opted to examine only the most upvoted answers so that we could analyze the characteristics of the answers that users deemed most favorable [48]. Accordingly, only one answer for each question was used from an average of 27.4 and 2.7 answers detected on Quora and Naver KiN, respectively. All data and source code files can be found in the repository.

**Figure 2:**
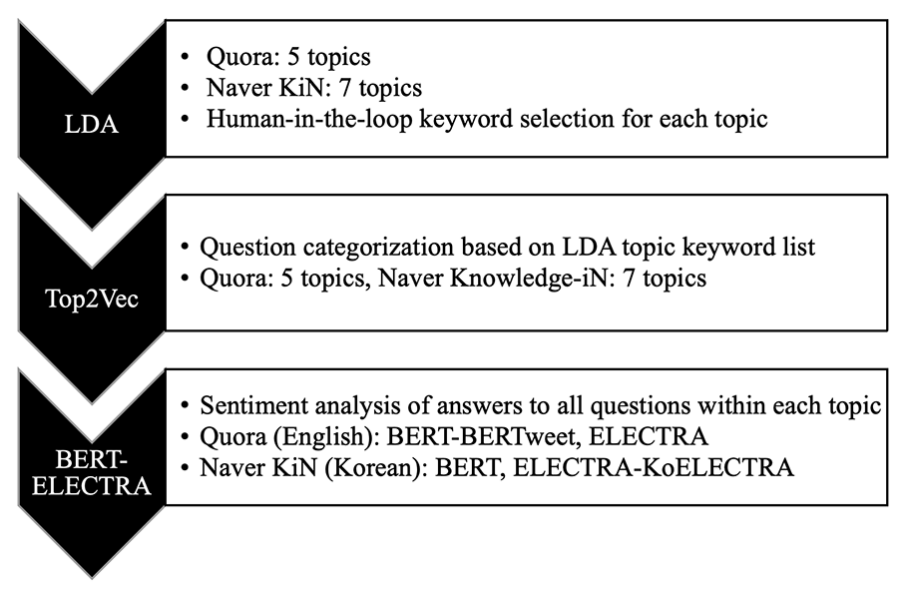
Overview of data analysis framework. Questions from Quora and Naver Knowledge-iN (Naver KiN) are grouped into topics using LDA and Top2Vec, with human-in-the-loop keyword selection. Sentiment analysis of answers within each topic is then performed using BERT- and ELECTRA-based models. BERT: Bidirectional Encoder Representations from Transformers; ELECTRA: Efficiently Learning an Encoder that Classifies Token Replacements Accurately; LDA: Latent Dirichlet Allocation.

In this study, we used 2 major approaches to analyze the questions and answers (Figure 2), including topic categorization for questions based on Latent Dirichlet Allocation (LDA) [57] and Top2Vec [58], and sentiment analysis for answers using ensemble-bagging with Bidirectional Encoder Representations from Transformers (BERT) [59] and Efficiently Learning an Encoder that Classifies Token Replacement Accurately (ELECTRA) [60]. For answers, this study used the most upvoted answers to focus on the characteristics of answers that users deemed most favorable. These natural language processing (NLP) techniques have been shown to be effective for analyzing large-scale data [61].

### Categorization of Questions: LDA and Top2Vec

In this study, we combined machine learning predictions with human-in-the-loop validation to categorize questions from Quora and Naver KiN. To this, we applied topic modeling to identify key topics across the documents, offering guidance for strengthening public health communication strategies [62]. Specifically, LDA was used to categorize the questions on Quora into 5 topics and those on Naver KiN into 7 topics. We initially extracted 50 topics, which is the optimal number of topics determined by the coherence score, and manually merged them into 5 topics for Quora and 7 topics for Naver KiN. Researchers (SY, SHJ, JC, and YJY) discussed and decided on each topic together. While merging topics, we discovered that Naver-KiN covered more topics than Quora.

LDA uses documents, words, and latent variables that represent the latent topics associated with word sequences. Although LDA is effective in restricting the number of topics and offering keywords associated with each topic, it does not quantify the similarity between each keyword and its topic (it mainly uses document-word frequency when categorizing topics). That makes it challenging to determine which words are most relevant to the topic when relying solely on human decisions for keyword selection. To address this limitation, we incorporate Top2Vec, which preserves word semantics by representing words and documents in a shared embedding space and derives topic representations directly from these semantic structures.

Building on this motivation, we adopt a hybrid LDA-Top2Vec pipeline to combine probabilistic topic structure with semantic embedding-based topic modeling. While Top2Vec is often applied directly to raw documents, we instead use LDA to first identify topic-relevant keywords, which serve as a coarse topical filter. This step constrains the semantic space Top2Vec explores, enabling it to focus on semantically meaningful regions associated with each topic. As a result, this hybrid approach yields more interpretable and stable topic representations than applying Top2Vec alone to the full document corpus. While formal coherence scores were not used as a selection criterion, this hybrid approach was chosen to prioritize interpretability and topic stability.

**Figure 3:**
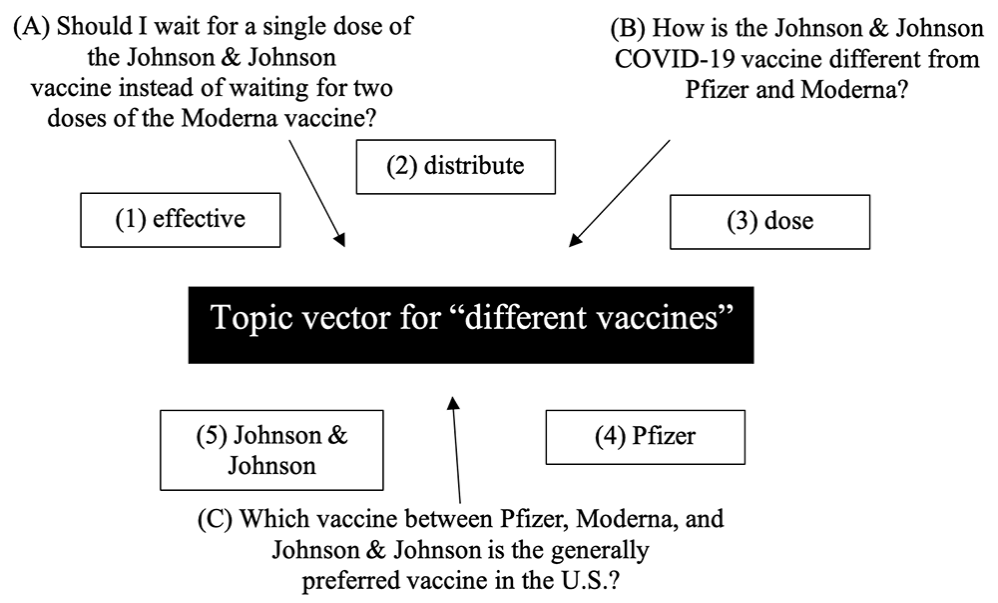
Illustration of topic vector construction for “different vaccines.” The topic vector is defined as the centroid of the document (question) vectors (A, B, and C) and the associated keywords (1, 2, 3, 4, and 5). Keywords that are semantically closest to this centroid are used to characterize the topic.

Operationally, the LDA-extracted keywords were represented as word vectors, and the questions were represented as document vectors in the vector space. Figure 2 illustrates the process of question analysis using the mixed LDA and Top2Vec method. Figure 3 further visualizes a semantic space that consists of word vectors (enclosed numbers 1, 2, 3, 4, and 5) and document vectors (labeled A, B, and C). The centroid of the document vectors is a topic vector (black central square labeled topic vector for different vaccines). The word vectors in Figure 3 are the words with the closest semantic relationship to the topic vector, whereas the LDA keywords indicate the word distribution per topic solely by frequency. In other words, LDA delineates the statistical relationship among topics, but it fails to capture semantic information about those topics [63].

### Sentiment Analysis of the Answers: Transformer-Based Models

Because COVID-19 greatly affected the sentiments expressed on social media [64], we used a document-level sentiment analysis to extract and classify perceptions and attitudes toward the topics discussed in the documents [32,65]. This analysis served as an indicator of public perception regarding COVID-19 concerns [66]. We used 2 transformer-based models, BERT and ELECTRA, that are capable of fine-tuning with pretrained models. The pretraining and fine-tuning mechanism enhances each model’s ability to effectively grasp the intricacies of downstream NLP tasks, such as sentiment analysis.

BERT is a widely used language model that uses pretrained data and a self-attention mechanism to generate contextualized vector outputs. It is applied to a range of downstream tasks, including sentiment analyses. BERT uses the masked language model; some tokens in the sentence are replaced with (MASK), and then the model is trained to regenerate the original tokens [59]. On the other hand, ELECTRA, which is another pretrained transformer model, uses a discriminator to predict the replaced tokens by generating token output instead of relying on a masked language model [60]. Both models have been widely used for various NLP downstream tasks [67], and we used models fine-tuned for an English sentiment analysis and a Korean sentiment analysis.

For the English language model, both BERT-based [68] and ELECTRA-based [60] were specifically fine-tuned for a sentiment analysis using Twitter datasets. While Twitter and Quora have posts of different lengths (a Twitter post consists of 8 words on average and a Quora post consists of 40 words on average) [69], both Twitter and Quora texts are social media data originating from public interactions on a social media platform. They share similar traits, and the model fine-tuned on Twitter was effectively applied to the Quora dataset [70], and we conducted a sentiment analysis of Quora data with 66% (33/50) accuracy. For the Korean language model, both BERT-based [71] and ELECTRA-based [72] were fine-tuned using Naver KiN shopping reviews, Steam game reviews, and movie reviews. We evaluated the accuracy of these models for Naver KiN data and achieved 72% (36/50) accuracy in the sentiment analysis. This choice thus mirrored the strategy we used for the English model.

One limitation of using transformer models is the constraint of the input tensor size, which is restricted to a length of 128 tokens. This limitation can sometimes introduce biases because only the first 128 tokens are considered, potentially leading to inaccurate sentiment analysis results. For instance, the beginning of a post might convey a positive sentiment by starting with a greeting, for example, that is not representative of the main content of the post. To address that issue, we divided each post into chunks of 128 tokens, measured the sentiment of each chunk, and then averaged the sentiment values of all the chunks to determine the overall sentiment result.

**Figure 4:**
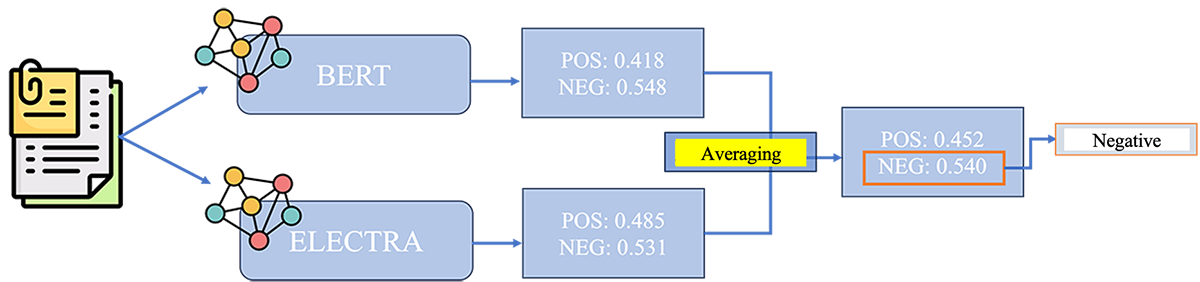
Ensemble averaging for sentiment analysis. Positive and negative sentiment scores are independently predicted using BERT and ELECTRA. The corresponding scores are then averaged across models, and the sentiment label with the higher averaged score is selected as the final prediction. BERT: Bidirectional Encoder Representations from Transformers; ELECTRA: Efficiently Learning an Encoder that Classifies Token Replacements Accurately; Neg: negative; POS: positive.

To further enhance the validity of the sentiment analysis, we used an ensemble method called the bagging or bootstrap aggregating technique, which is known for its effectiveness in classification tasks. This method generates an ensemble classification output that outperforms the accuracy of a single prediction value [73,74]. We applied it to predict improved sentiment values for each answer using both BERT and ELECTRA. An overview of the process is provided in Figure 4. The outcomes from both models were the probabilities of positive and negative sentiments. Subsequently, we computed the average probabilities of positive and negative sentiments. Once we obtained the averaged positive and negative probabilities, we compared them and used the higher one.

### Ethical Considerations

Ethical review was not required for this study because it exclusively used publicly available social media content shared in accordance with platform policies. As the data were publicly accessible, Institutional Review Board approval and a waiver of informed consent were not required [75,76]. This study did not use or infer individuals’ identities but instead examined vaccine-related opinions at an aggregate level to provide an overall picture of public perceptions. To protect user privacy, all identifiable information was removed during preprocessing, and all questions and comments presented in this manuscript were paraphrased. The final aggregated dataset contains no personally identifiable information and cannot be traced back to individual users.

## Results

### Topic Analysis of Questions

Table 1 presents the topic modeling results for Quora, where 5 primary topics were identified. The most frequently discussed topic was different vaccines (978/3952, 25%), with users asking about characteristics and comparisons of various vaccine brands such as AstraZeneca (AstraZeneca plc) and Moderna (Moderna, Inc). This was followed by government policy (821/3952, 21%), where questions centered on administrative decisions and vaccine distribution, with keywords including “require,” “Trump,” and “supply.” Variants (782/3952, 20%) included discussions focused on the severity of new COVID-19 strains and vaccine efficacy. The topic of visiting overseas (743/3952, 19%) comprised questions about travel eligibility based on vaccination status. The least frequent topic, effects of vaccine (626/3952, 16%), involved concerns about immunity, side effects, and infection risks.

Table 2 shows the topic modeling outcomes for Naver KiN, which yielded 7 topics. The most common was effects of vaccine (790/3952, 20%), marked by terms like “side effects” and “uptake,” where users expressed hopes for effective vaccination and sought information on its impact. Vaccination appointments followed (711/3952, 18%), with users asking about eligibility and scheduling logistics. The variants topic (592/3952, 15%) reflected interest in vaccine efficacy against emerging strains. Government policy (553/3952, 14%) included questions about national vaccination strategies. School and education (513/3952, 13%) highlighted questions about children’s school attendance in relation to family vaccination status. Visiting overseas (474/3952, 12%) addressed international travel feasibility amid varying vaccination policies. The least common topic was different vaccines (316/3952, 8%), where users asked about vaccine suitability for individual health conditions.

**Table 1:** Example question for each topic in Quora. For each topic identified in the Quora dataset, the table reports the proportion of questions assigned to the topic and provides an example question illustrating the topic content (N=3952).

Label	Topic proportion, n (%)	Example question
Different vaccines	978 (25)	“How come the other vaccines, including the Pfizer and Moderna vaccines, require 2 doses; however, the Johnson & Johnson COVID- 19 vaccine requires only one dose?”
Government policy	821 (21)	“On his tonight show, Sean Hannity credits Donald Trump with developing the COVID-19 vaccine rather than President Biden. Who in your opinion deserves the credit?”
Variant	782 (20)	“Does the delta variant of COVID-19 pose the highest threat to individuals who have not yet received vaccinations because it is more contagious, causes a more serious illness, and is not affected by the current vaccines?”
Visiting overseas	743 (19)	“Would you get vaccinated only to travel if many of the largest airlines in the world require verification of vaccination status in your passport and prevent unvaccinated people from traveling?”
Effects of vaccine	626 (16)	“Is it acceptable to state that you do not want the vaccine because everyone you know who has received the COVID-19 vaccine has become ill shortly after vaccination?”

**Table 2:** Example questions for each topic in Naver Knowledge-iN (Naver KiN). For each topic identified in the Naver KiN dataset, the table reports the proportion of questions assigned to the topic and provides an example question illustrating the topic content (N=3952).

Label	Topic proportion, n (%)	Example question
Effects of vaccine	790 (20)	“I heard that individuals under 18 years of age cannot be vaccinated but why? I also want to get vaccinated. COVID-19 will come to an end if everyone gets vaccinated quickly and becomes immune to the virus. I want to take off my mask and go somewhere.”
Vaccine appointment	711 (18)	“Can civil defense corps members also get vaccinated? When are they eligible to get vaccinated? Tell me the vaccination schedule for both the 1st and 2nd dose.”
Variant	592 (15)	“They say that COVID-19 vaccines are effective but is it too early to come to conclusions? For vaccines to be effective, antibodies should last for a long period. However, for COVID-19 vaccines, antibodies do not last even 1 year, indicating they are effective only for a short period of time. (…) Thus, is it too early to determine the effectiveness of the vaccines?”
Government policy	553 (14)	“I have recently taken interest in our government. I believe our government has responded well to the COVID-19 outbreak compared with other countries, and the delay in vaccine supply appears reasonable because vaccines are prioritized to countries that have not managed the virus well. (…) However, why are there so many negative comments regarding the government on Naver KiN? I am just wondering. Maybe I am wrong but I am leaving a comment because I want to know why people are so angry.”
School and education	513 (13)	“My parents were vaccinated. Can I go to school?”
Visiting overseas	474 (12)	“How serious is the COVID-19 situation in Japan? Has Japan started vaccinations? Please tell me specifically about this situation. I am planning on traveling to Japan and was wondering whether it would be possible to go to Japan after COVID-19 comes to an end. I am planning my trip for next year. Will COVID-19 end in both countries by then?”
Different vaccines	316 (8)	“I am in my 20s and I have hypothyroidism. Are people with hypothyroidism considered chronically ill patients? When can people with chronic diseases get vaccinated? Which vaccine will I receive?”

### Sentiment Analysis of Answers

**Figure 5:**
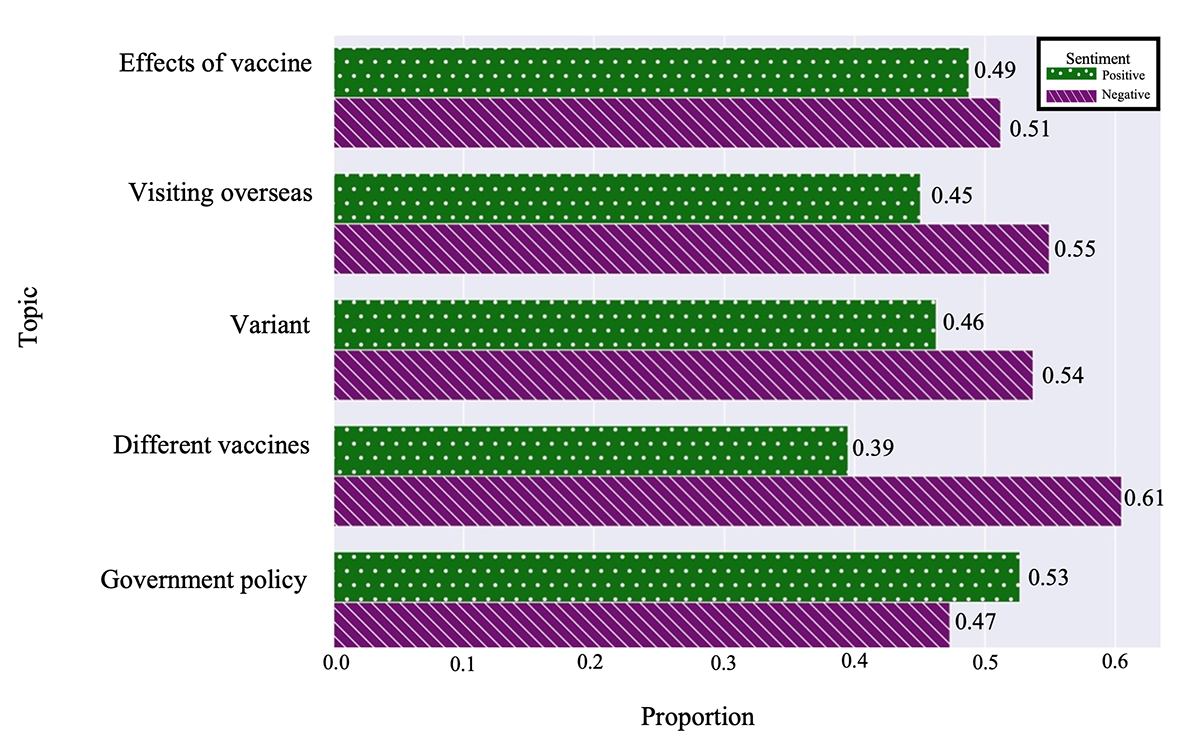
Sentiment proportions per topic in Quora.

A sentiment analysis was used to examine the answers given on both Quora and Naver KiN. Figure 5 illustrates the proportions of sentiments in Quora answers to questions from each topic. Across 4 of the topics—effects of vaccine, visiting overseas, variants, and different vaccines—the number of answers with negative sentiment exceeded the number of positive ones. However, within the topic of government policy, answers with positive sentiment were more prevalent than negative ones.

In Figure 5, positive versus negative sentiments were observed as follows: effects of vaccines (306/626, 48.79% vs 320/626, 51.12%), visiting overseas (335/743, 45.05% vs 408/743, 54.95%), variants (362/782, 46.29% vs 420/782, 53.71%), different vaccines (386/978, 39.47% vs 592/978, 60.53%), and government policy (437/821, 53.23% vs 384/821, 46.77%), where the first value represents positive sentiment and the second represents negative sentiment.

**Figure 6:**
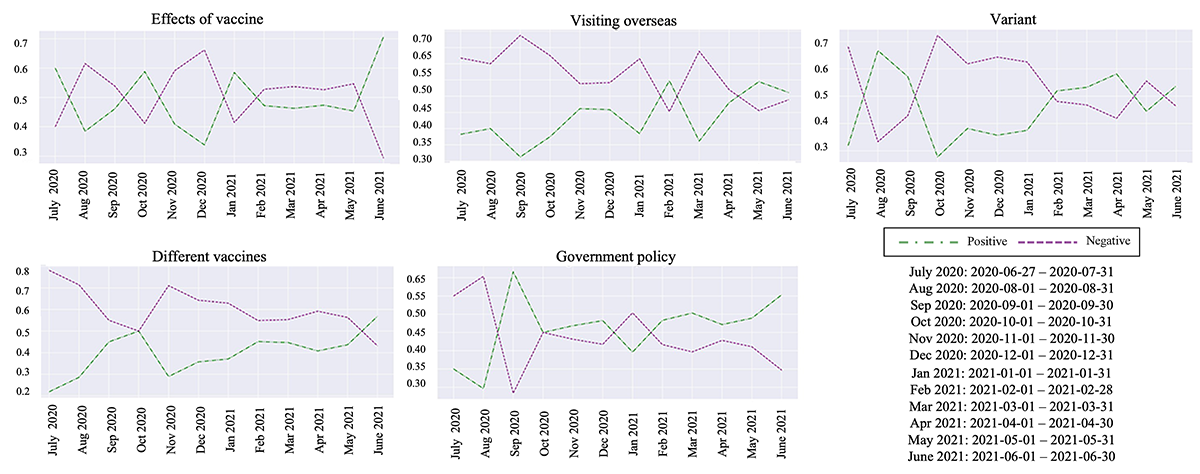
Temporal analysis of sentiments per topic in Quora. Due to limited data availability, 4 days at the end of June 2020 were merged into July 2020 for the analysis.

We analyzed sentiment outcomes on a monthly basis from June 2020 to June 2021, focusing on topic popularity over time (see Figure 6). While finer-grained temporal analyses are possible, we adopted monthly aggregation to reduce sparsity and short-term volatility and to emphasize broader discourse trends rather than reactions to specific events. Across all topics except different vaccines, positive-negative sentiment crossovers occurred at least 3 times. After May 2021, positive sentiment exceeded 50%, indicating its dominance across topics. Notably, only government policy consistently showed a surplus of positive sentiment over negative for 4 consecutive months after February 2021.

During this period, the vaccination rate increased by 15.3%, from 17.45% to 32.78% [52], coinciding with heightened public attention to vaccine rollout and access. Positive sentiment toward government policy appears to reflect evaluations of these concrete policy outcomes rather than a broader shift in institutional trust. In particular, the temporary increase in positive sentiment after February 2021 aligns with major administrative transitions and policy announcements, including changes in federal coordination, alongside continued public recognition of vaccine development efforts initiated under Trump, as reflected in the example in Table 1.

Prior research similarly suggests that during public health crises, shifts in public trust toward government are closely tied to public perception of policy responses and outcomes. Notably, policy-specific achievements (eg, visible progress in crisis management) are often associated with temporary increases in positive evaluations even amid persistent skepticism toward government institutions [77]. Thus, the observed temporal positivity in “government policy” sentiment likely captures situational approval of pandemic response measures rather than a reversal of widespread distrust in US governance.

**Figure 7:**
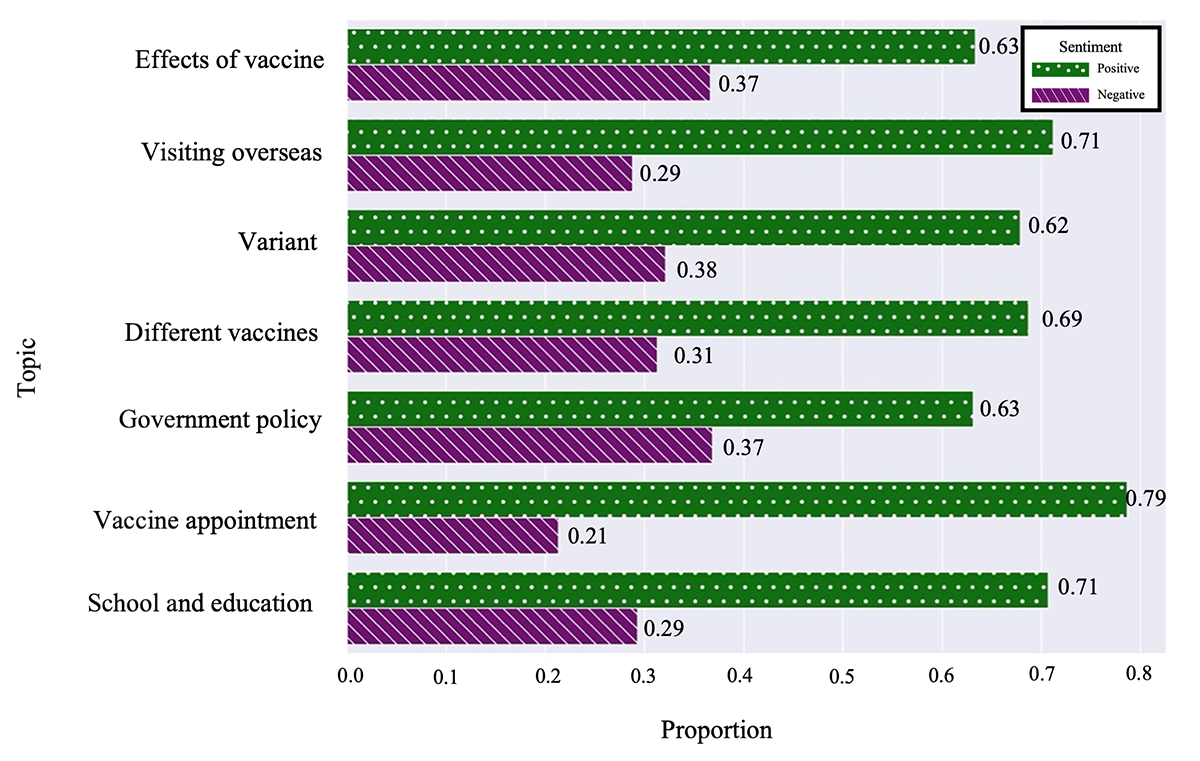
Sentiment proportions per topic in Naver Knowledge-iN (Naver KiN).

**Figure 8:**
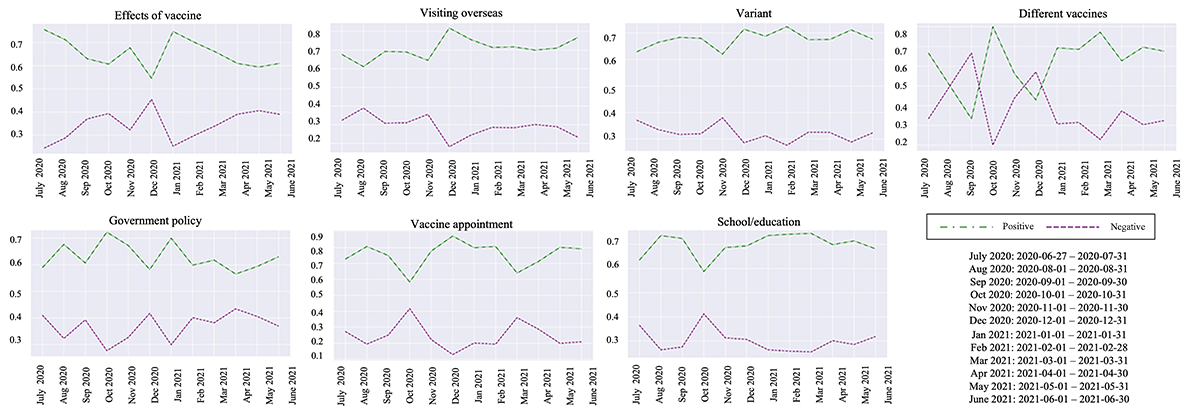
Temporal analysis of sentiment per topic in Naver KiN. Due to limited data availability, 4 days at the end of June 2020 were merged into July 2020 for the analysis.

The results of the sentiment analysis of answers to questions on Naver KiN are depicted in Figure 7. Across all 7 topics—different vaccines, government policy, schools and education, vaccine appointments, variants, effects of vaccines, and visiting overseas—positive sentiments were predominant over negative sentiments. This overarching trend is also highlighted in Figure 8, which shows that positive sentiment consistently exceeded 50% throughout the year except for the topic of different vaccines.

In Figure 7, the proportions of positive versus negative sentiment were as follows: effects of vaccines (498/790, 63.04% vs 292/790, 36.96%), visiting overseas (337/474, 71.10% vs 137/474, 28.90%), variants (367/592, 61.99% vs 225/592, 38.01%), different vaccines (218/316, 68.99% vs 98/316, 31.01%), government policy (348/553, 62.93% vs 205/553, 37.07%), vaccine appointment (562/711, 79.04% vs 149/711, 20.96%), and school and education (364/513, 70.96% vs 149/513, 29.04%).

For the topic of different vaccines, 4 positive-negative sentiment crossovers occurred between June 2020 and January 2021, often driven by concerns about side effects and individual health conditions. During this period, confirmed COVID-19 deaths per million rose sharply by 313% [52]. As shown in Table 2, a question regarding vaccine eligibility for individuals with chronic illnesses received a relevant response. After February 2021, coinciding with the launch of South Korea’s vaccination program on February 26, positive sentiment prevailed, with no further crossovers observed [52].

### Sentiment Analysis of Q&A Pairs

After analyzing the sentiments of the answers, we examined question–answer sentiment pairs within each topic to see if the question’s sentiment might affect the answer. Table 3 shows the sentiments of question-answer pairs on Quora. In 4 topics—effects of vaccine, visiting overseas, variants, and different vaccines—the predominant sentiment pairs are negative-negative, followed by negative-positive, positive-positive, and positive-negative. For the topic of government policy, negative-positive sentiment pairs are as prevalent as negative-negative, with positive-positive and positive-negative following.

Table 4 illustrates the sentiment for question-answer pairs on Naver KiN. Across all topics, positive-positive and negative-positive pairs constituted more than 63% of the sentiment. For 6 topics—effects of vaccines, visiting overseas, different vaccines, government policy, vaccine appointments, and school and education—positive-positive pairs were the most prevalent, followed by negative-positive pairs. In contrast, for the topic of variants, negative-positive pairs were the most prevalent, followed by positive-positive pairs.

**Table 3:** Question-answer sentiment pairs for each topic on Quora.

Topic	Positive-positive, n (%)	Positive-negative, n (%)	Negative-positive, n (%)	Negative-negative, n (%)
Effects of vaccines (N=626)	101 (16.07)	49 (7.85)	205 (32.71)	271 (43.36)
Visiting overseas (N=743)	84 (11.3)	68 (9.11)	251 (33.75)	340 (45.84)
Variants (N=782)	123 (15.78)	91 (11.61)	239 (30.51)	329 (42.11)
Different vaccines (N=978)	114 (11.66)	88 (8.97)	272 (27.86)	504 (51.52)
Government policy (N=821)	123 (14.87)	73 (8.78)	314 (37.82)	311 (37.53)

**Table 4:** Question-answer sentiment pairs in each topic on Naver Knowledge-iN (Naver KiN).

Topic	Positive-positive, n (%)	Positive-negative, n (%)	Negative-positive, n (%)	Negative-negative, n (%)
Effects of vaccines (N=790)	279 (35.32)	187 (23.67)	219 (27.72)	105 (13.29)
Visiting overseas (N=474)	239 (50.42)	101 (21.31)	98 (20.68)	36 (7.59)
Variants (N=592)	178 (30.07)	102 (17.23)	189 (31.92)	123 (20.78)
Different vaccines (N=316)	125 (39.56)	49 (15.51)	93 (29.43)	49 (15.5)
Government policy (N=553)	177 (32.01)	90 (16.27)	171 (30.92)	115 (20.8)
Vaccine appointment (N=711)	374 (52.6)	110 (15.47)	188 (26.44)	39 (5.49)
School and education (N=513)	207 (40.35)	85 (16.57)	157 (30.61)	64 (12.47)

## Discussion

### Principal Findings

Social media provides valuable insights for policymakers developing health policies [27]. Our study pioneers exploring public discourse on vaccines in 2 countries using advanced NLP techniques, surpassing traditional survey-based methods. This approach reveals hidden aspects of vaccine perceptions beyond conventional demographics. We analyzed overall perceptions (via topic analysis) and communication within each topic (via sentiment analysis). In this discussion, we consider platform characteristics and acknowledge the study’s limitations.

### Comparative Discourse on the COVID-19 Vaccine

The United States and Korea both have concerns about 5 topics, including effects of the vaccine, visiting overseas, variants, different vaccines, and government policy; however, Korea uniquely highlights vaccine appointments and school and education. Vaccine appointments became a significant issue in Korea due to delays in vaccine distribution [49], with the government implementing an age-based distribution policy to respect the cultural emphasis on older adults and their high risk of serious disease. Although prioritizing the older adults might have effectively reduced COVID-19–related mortality and morbidity within this age group, other vulnerable populations, such as prisoners and the homeless, experienced relative disadvantages. Therefore, the equitable distribution of vaccinations is a topic requiring open discussion.

Naver KiN also revealed a unique topic about school and education that did not appear on Quora. This topic highlights how deeply education is valued in Korean culture, which made vaccinating school-aged children and their parents crucial [78]. This contrasts with the United States, where, despite the CDC’s recommendations [79], vaccine hesitancy remained a notable barrier among many parents [80]. Thus, the Naver KiN results underscore that cultural values and societal priorities, such as Korea’s strong commitment to educational success, played a significant role in shaping public attitudes toward COVID-19 vaccination.

Based on those topics, we investigated the sentiments expressed in question-answer pairs and gleaned insights into the public perceptions of COVID-19 vaccines expressed on social Q&A sites in the United States and Korea. Our findings highlight distinct sentiment patterns reflective of the cultural and informational contexts of these countries. Quora and Naver KiN both have a similar tendency of consistency between a question’s sentiment and the answers it receives (eg, positive [Q]-positive [A] or negative [Q]-negative [A]). However, users of the 2 platforms expressed different sentiments toward the COVID-19 vaccine.

The sentiment analysis of Quora data reveals that in the United States, negative sentiment was predominant in discussions about the effects of the vaccine, visiting overseas, variants, and different vaccines. In those topics, negative-negative (question-answer) pairs are the most common, indicating widespread skepticism and concern among users [15,25]. Unlike Quora, the majority of answers were positive in the Naver KiN data from Korea, with positive-positive (question-answer) and negative-positive pairs constituting more than 63% of the total. This difference may be attributed to varying levels of trust in the government between the 2 countries.

At the time we collected data from Naver and Quora, South Korea was effectively managing the pandemic, and public trust in the government was high, consistent with previous research showing high compliance with COVID-19 government policies [81]. As a result, both questions and answers were generally positive. The only topic that saw a higher occurrence of negative questions paired with answers with positive sentiment was related to variants, likely because the Korean government could not control the emergence of variants, leading to more negative sentiment. Even in discussions about variants, where there was more negative sentiment toward the question, the sentiment toward the answers remained largely positive, indicating resilience and hope [82,83]. In contrast, in the United States, the situation was markedly different. Public trust in the US government was lower, and adherence to COVID-19 policies was less consistent [81]. The skepticism and concerns expressed in the US discussions may have been driven by widespread dissatisfaction with how the pandemic was being handled, leading to a pervasive negative sentiment across various topics [81-83].

These contrasting attitudes suggest that the differences observed in the public discourse on the COVID-19 vaccine in the United States and South Korea may stem from cultural and situational factors rather than true variations in public perceptions. Given that the unique social context of each country shapes public discourse and sentiment, it can be inferred that communication strategies targeted to cultural contexts are required to address vaccine hesitancy. In the United States, public health campaigns should counter misinformation [32] and address specific safety concerns, engaging trusted community leaders and positive narratives about vaccination benefits. In Korea, on the other hand, maintaining positive public discourse through transparency and timely updates is crucial. The unique focus on vaccine appointments and education in Korean discussions highlights specific information needs that should be addressed.

### Effects of the 2 Platforms on Sentiment

Platform-specific user dynamics also play a role in shaping public discourse [84,85], affecting the sentiments of each platform’s answers. The presence of health care professionals on Naver KiN, who provide expert opinions, likely contributes to the positive sentiment and higher likelihood of their answers being perceived as trustworthy, in contrast to the diverse user base on Quora. Overall, understanding and leveraging these distinct sentiment patterns can help public health authorities craft more effective communication strategies to promote a positive and informed discourse about COVID-19 vaccination.

To select the responses for our analysis, we used a methodology intended to mirror the typical user experience on these platforms. Responses were chosen based on specific criteria designed to reflect a balance between popularity and relevance and, above all, to reflect which answers the users deemed most effective. This approach aligns with methodologies used in previous user-generated content analyses on platforms such as Quora and Naver KiN [48].

### Limitations of This Study

This study has several limitations. First, there was a notable variance in the average length of answers between Quora and Naver KiN. This discrepancy might have introduced potential bias when comparing the answers. Future studies could mitigate this by standardizing the length of answers or selecting questions with a similar number of words for a more meaningful comparison. In addition, platform-specific user demographics may partially confound cross-cultural comparisons. Naver KiN serves as a dominant information-seeking platform in South Korea, characterized by large-scale daily participation and broad distribution of user expertise [48,86]. In contrast, Quora users in the United States tend to be younger and more educated [87]. These structural differences may shape how questions are shaped, how answers are evaluated, and how sentiment is expressed. As a result, the observed sentiment asymmetry between the 2 platforms may reflect platform participation norms and demographic composition, as well as national-level attitudes.

Second, although we used models already trained on our target social media outlets, Quora and Naver KiN, we applied both topic modeling and sentiment analysis models without fine-tuning them with our data. This may result in less optimal and lower-performing outputs from the experiments. To mitigate this limitation, fine-tuning each model should be performed to yield more reliable results in future studies.

Third, our analysis focuses on the most favorable answers associated with each question. This design choice may limit the generalizability of findings regarding public perception. By prioritizing community-validated information, often in the form of expert-driven responses, this analysis may also introduce a positive bias toward answers. Thus, the observed association between question sentiment and answer sentiment should be interpreted as reflecting how question framing relates to the community-validated information, rather than the full range of public perception, which may include greater uncertainty, disagreement, or negative affect.

Finally, we acknowledge as a limitation that no explicit bot-detection or automated content-authenticity filtering algorithm was applied. Given the highly politicized and time-sensitive nature of vaccine-related discourse during 2020-2021, bot-generated content may have contributed to the observed sentiment patterns. Although we partially mitigated this risk by excluding unanswered questions and constraining questions that include the keyword “vaccine” (in Korean, 백신), these measures did not guarantee the removal of automated or coordinated accounts. As such, some sentiment estimates may reflect amplified or artificial signals rather than purely user opinions.

### Conclusions

This study has provided critical insights into the dynamics of vaccine discourse, underscoring the need for context-specific strategies for promoting vaccination. By melding state-of-the-art NLP technologies with a cross-cultural analysis, we have forged a path toward more informed, efficacious, and targeted efforts in public health communication, both in the ongoing battle against the COVID-19 pandemic and beyond. Above all, this study underscores the paramount importance of harnessing social media as a prolific source of public sentiment and information needs, particularly within the sphere of vaccine hesitancy. Analyzing public discourse on platforms such as Quora and Naver KiN provides researchers with an unfiltered view of individuals’ concerns and perceptions, offering insights invaluable for crafting responsive public health strategies.

Our findings reaffirm the profound effects of cultural and societal factors on public attitudes toward vaccines. The stark contrast observed between the United States and Korea in terms of vaccine perceptions underscores the need for culturally sensitive approaches in health communication and vaccination campaigns. This study advances the academic landscape by demonstrating the efficacy of NLP technologies for comprehending public perceptions and information needs related to vaccines. NLP methods transcend traditional survey methodologies, providing a more naturalistic perspective rooted in real-world discussions on community-driven social media platforms. Our innovative amalgamation of LDA, Top2Vec, and BERT- and ELECTRA-based sentiment analyses sets a pioneering benchmark for future research in this domain. This multifaceted approach augments our comprehension of the intricacies encircling vaccine discourse and can be adapted to explore analogous public health issues across diverse countries and cultures.

As for practical insights, our findings have implications for policymakers and health communication practitioners in both countries. The discernment of disparities in vaccine-related information needs and perceptions between the United States and Korea underscores the necessity of tailoring public health campaigns to the specific nuances of each nation’s context. Effectively addressing vaccine hesitancy—a complex interplay of safety concerns, access barriers, and misinformation—demands a nuanced, multifaceted approach. Our study underscores the need for policy interventions that take these underlying factors into account, while also recognizing the profound influence of cultural and societal values on vaccination decisions.

## Appendix

Multimedia Appendix 1 STROBE checklist.

## Abbreviations

BERT: Bidirectional Encoder Representations from Transformers
CDC: Centers for Disease Control and Prevention
ELECTRA: Efficiently Learning an Encoder that Classifies Token Replacement Accurately
H1N1: Hemagglutinin Type 1 and Neuraminidase Type 1
LDA: Latent Dirichlet Allocation
MERS: Middle East Respiratory Syndrome
Naver KiN: Naver Knowledge-iN
NLP: natural language processing
Q&A: question and answer
STROBE: Strengthening the Reporting of Observational Studies in Epidemiology
WHO: World Health Organization

## Data Availability

The data that support the findings of this study are openly available in GitHub repository.
